# DAR-Net: Dense Attentional Residual Network for Vehicle Detection in Aerial Images

**DOI:** 10.1155/2021/6340823

**Published:** 2021-11-26

**Authors:** Kaifeng Li, Bin Wang

**Affiliations:** School of Communication and Information Engineering, Shanghai University, Shanghai, China

## Abstract

With the rapid development of deep learning and the wide usage of Unmanned Aerial Vehicles (UAVs), CNN-based algorithms of vehicle detection in aerial images have been widely studied in the past several years. As a downstream task of the general object detection, there are some differences between the vehicle detection in aerial images and the general object detection in ground view images, e.g., larger image areas, smaller target sizes, and more complex background. In this paper, to improve the performance of this task, a Dense Attentional Residual Network (DAR-Net) is proposed. The proposed network employs a novel dense waterfall residual block (DW res-block) to effectively preserve the spatial information and extract high-level semantic information at the same time. A multiscale receptive field attention (MRFA) module is also designed to select the informative feature from the feature maps and enhance the ability of multiscale perception. Based on the DW res-block and MRFA module, to protect the spatial information, the proposed framework adopts a new backbone that only downsamples the feature map 3 times; i.e., the total downsampling ratio of the proposed backbone is 8. These designs could alleviate the degradation problem, improve the information flow, and strengthen the feature reuse. In addition, deep-projection units are used to reduce the impact of information loss caused by downsampling operations, and the identity mapping is applied to each stage of the proposed backbone to further improve the information flow. The proposed DAR-Net is evaluated on VEDAI, UCAS-AOD, and DOTA datasets. The experimental results demonstrate that the proposed framework outperforms other state-of-the-art algorithms.

## 1. Introduction

Object detection, as an important topic in computer vision, aims to precisely localize the targets in given images and classify each target. This topic is of broad interest for potential applications of face detection, pedestrian counting, automatic driving, vehicle detection, etc. [1].

Before the emergence of deep learning, most traditional object detection algorithms which are based on hand-crafted features can be roughly divided into three steps: region selection, feature vector extraction, and region classification. In the region selection step, the input image is usually scanned by multiscale sliding windows to find the locations which may contain targets. These locations selected in the region selection step are called candidate regions. During the feature extraction step, low-level visual descriptors such as SIFT [2], HOG [3], or SURF [4] are used to extract and encode semantic information from each candidate region. In the final step, the encoded feature vectors of each candidate region are classified by classifiers such as SVM [5]. Although object detection algorithms based on traditional manual features have made some breakthroughs in detection accuracy, there still are two nonignorable limitations. Firstly, they inevitably generate many redundant candidate regions during region proposal steps, which leads to imbalanced class distribution during region classification steps. Secondly, hand-crafted feature extraction algorithms are not capable of capturing high-level semantic information; besides the low-level information it extracted is not sufficient for complex localization and classification problems. Because of these limitations, traditional object detection algorithms are generally time-consuming and inaccurate.

Recently, with the development of computer science and hardware technologies, as well as abundant data resources, more and more researchers give their top research priority to deep learning based object detection algorithms. Generally speaking, deep learning based object detection algorithms can be classified into two categories: two-stage detection algorithms, such as R-CNN families [6–9], FPNs [10–12], and their variants [13–15], and one-stage detection algorithms, such as YOLO families [16–19], SSD families [20, 21], and their variants [22–24]. Similar to traditional object detection algorithms, two-stage detection algorithms also contain region selection and region classification steps. However, different from traditional detection algorithms, two-stage detection algorithms utilize convolutional neural networks (CNN) to generate hierarchical feature maps including high-level semantic information in the feature extraction step. Without the region selection step, one-stage algorithms directly detect targets in different locations by bounding box regression processes and classification processes. Usually, two-stage algorithms achieve better results on benchmark detection tasks, while one-stage algorithms can achieve faster processing speed.

Powered by advanced remote sensing technologies and wide usages of UAVs, vehicle detection in aerial images, as a downstream research direction of object detection, becomes indispensable in many important applications such as disaster relief, population density estimation, parking lot planning, traffic monitoring, etc. However, the algorithms detecting targets in ground view images cannot be directly utilized for vehicle detection in aerial images because of the following differences between ground view images and aerial images:

1. Generally speaking, aerial images cover much larger areas than ground view images, while the sizes of targets in aerial images are much smaller than the sizes of targets in ground view images. This feature produces great difficulties for extracting information, especially spatial information, of targets from aerial images, which leads to further difficulties in correctly localizing the targets.
2. Due to the vertical shooting angle of aerial images, the texture of vehicles in aerial images is relatively simple. As a result, the background objects such as buildings are easily confused with the vehicles. In addition, the vehicles in aerial images usually appear with arbitrary orientations. These special characteristics make it difficult to correctly classify the targets.
3. The number of targets in aerial images is normally more than the number of targets in ground view images, which also brings difficulties to detect targets.

Considering the difficulties illustrated above, a new one-stage vehicle detection framework for aerial images is proposed in this paper. The main contributions of this paper are listed as follows:

1. A novel residual block, named dense waterfall residual block (DW res-block), is proposed in this paper. In each DW res-block, the partial information of each convolutional layer is transmitted into all subsequent layers; in other words, each layer can obtain the partial information from all preceding layers. Because of this design, the proposed framework can preserve the low-level spatial information and extract high-level information simultaneously during the feature extracting stage.
2. A multiscale receptive field attention (MRFA) module is proposed and plugged into the proposed DW res-block. The proposed MRFA module generates the multiscale receptive field feature maps by using dilated convolution and fuses these feature maps with the attention-weighted feature maps. The proposed attention module selects the informative feature from the feature maps and enhances the ability of multiscale perception of the proposed framework.
3. Utilizing the DW res-block and the MRFA module, a backbone for vehicle detection in aerial images is proposed. The proposed backbone extracts the semantic information from high-resolution feature maps to alleviate the loss of spatial information. Besides, downprojection units and transition layers are also employed in the proposed backbone to reduce the impact of information loss caused by downsampling and improve the information flow of the proposed framework, respectively.

## 2. Related Works

Due to the rapid improvement of deep learning and the wide utilization of UAVs in recent years, many CNN-based detection algorithms for aerial images have been proposed. Some typical algorithms are reviewed in this section.

In 2017, to solve the limitations of directly using Faster R-CNN for vehicle detection in aerial images, T. Tang et al. proposed an improved detection framework based on Faster R-CNN [25]. In that paper, T. Tang et al. designed a hyperregion proposal network (HRPN) to extract the target information from the fusions of hierarchical feature maps.

In 2018, Terrail J. et al. proposed a new framework based on Faster R-CNN, called Faster Rotation Equivariant Regions CNN (Faster RER-CNN) [26]. In that paper, Terrail et al. applied simultaneous orientation inference to the Faster R-CNN framework and proposed a new metric for measuring the performance of rotating object detection algorithms. Yang MY et al. proposed a new double focal loss convolutional neural network framework (DFL-CNN) in [27]. The DFL-CNN framework combined the low-level features and high-level features by using skip-connection. Besides, Yang MY et al. also adopted focal loss to DFL-CNN to solve the problem caused by imbalanced numbers of positive and negative targets. Rotation Dense Feature Pyramid Networks (R-DFPN) were proposed in [28] to solve the difficulties caused by complex backgrounds and intensive targets in aerial images. In that paper, Yang X. et al. designed a dense connection network based on Feature Pyramid Networks (FPN) to extract multiscale features containing high-level semantic information. To build strong feature maps, Azimi et al. proposed a novel joint image cascade and feature pyramid network (ICN and FPN) in [29]. The proposed algorithm also used rotation region proposals to improve the location accuracy of object detection in aerial images.

In 2019, Pang J. et al. proposed a remote sensing region-based convolutional neural network (R2-CNN) [30] for small object detection in aerial images. In that paper, a new residual structure called Tiny-Net containing a global attention block was designed to suppress false positives caused by objects belonging to the background. Li C. et al. proposed a learning objectwise semantic representation for object detection in aerial images in [31]. In the proposed algorithm, proposal detection was guided by using a semantic segmentation module. Mandal et al. proposed a one-stage vehicle detection network (AVDNet) for small vehicle detection in aerial images in [32]. In the AVDNet paper, ConvRes residual blocks were designed to retain fine-grained feature in deep convolutional layers.

In 2020, Wu et al. proposed a novel geospatial object detection framework, called Fourier-based rotation-invariant feature boosting (FRIFB) [33]. In that paper, the rotation-invariance FourierHOG, ACF, FPGM, and boosting learning were integrated to achieve an effective and robust framework. The corresponding rotation-invariant channel maps were obtained by the FourierHOG algorithm and subsequently refined by ACF against object rotation and shift. By performing extensive experiments, it can draw a conclusion that the proposed method is robust to objects rotation. Shen et al. proposed a lightweight deep convolutional network for vehicle detection in aerial detection [34]. In that paper, a new aerial vehicle image dataset was also published. Zhou et al. proposed an anchor-free polar remote sensing object detector (P-RSDet) [35]. In that study, the author used a polar coordinate system for arbitrary-oriented object detection rather than Cartesian coordinates. To make algorithms gaze at the targets in an image, Chen et al. proposed a novel multiscale spatial and channelwise attention (MSCA) mechanism [36]. MSCA paid more attention to the spatial area and the feature channel related to the foreground. Furthermore, MSCA can be easily plugged into classic deep learning based detection frameworks. Wang B. et al. proposed an Improved FBPN Based Detection Network for small object detection in aerial images. In that paper, an improved feature-balanced pyramid network (FBPN) [37] was designed to balance the high-level and low-level feature maps.

In 2021, Yi et al. proposed an oriented keypoint-based detection framework to solve the class imbalance problem of anchor-based detection algorithms [38]. In that paper, the horizontal keypoint-based detection algorithm was improved to the oriented keypoint-based object detection framework and the box boundary-aware vectors (BBAVector) were proposed to describe the oriented bounding box. The experiment proved that BBAVectors can achieve better performance of object detection in aerial images. Li et al. proposed an efficient detection framework called simple convolutional neural networks (simple-CNNs) in [39], which can be directly applied to real-world applications. In that paper, a new loss function, namely, the change-IOU Loss (CI-Loss), was designed to improve the detection performance with the target position information.

## 3. Methods

**Figure 1:**
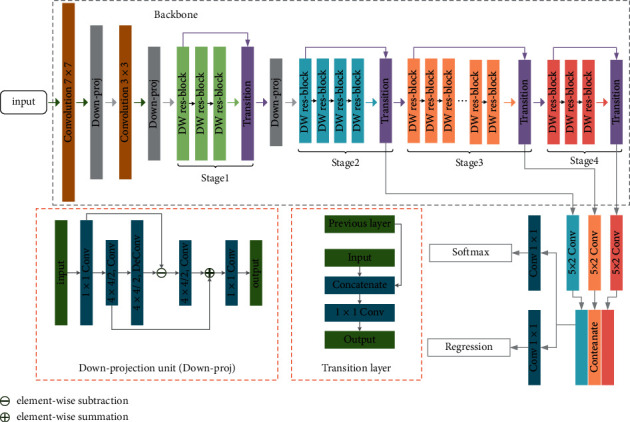
The overall architecture of the proposed framework.

To deal with complicated vision-based applications, researchers prefer to increase the depth of convolutional neural networks to get stronger power of information perception and learning ability. However, before various residual blocks have been proposed, the maximum depth of the mainstream convolutional neural networks at that time is restricted to relatively small numbers because of the problem of degradation problem. For example, Alex-Net [40] has 7 3×3 convolutional layers, VGG-Net [41] has 16 or 19 3×3 convolutional layers, and Google-Net [42] has 22 3×3 convolutional layers. In 2016, K. He et al. proposed the residual networks (ResNets) [43] which stack multiple residual blocks and construct the identity mapping by shortcuts. This design can effectively solve the degradation problem, which leads to a much deeper network (e.g., 53 or 101 3×3 convolutional layers) [18, 43] comparing to other kinds of convolutional neural networks. Driven by various residual blocks, various deep learning based vision algorithms develop rapidly, especially in the research fields of object detection. However, in the research field of vehicle detection in aerial images, the state-of-the-art residual networks have their limitations. Because the scales of the target sizes are significantly smaller and the background areas are larger and more complicated than normal object detection, high-level semantic information and low-level spatial information are equally important for vehicle detection in aerial images. Although deeper residual networks do have the advantages in exploring deeper features that contain rich semantic information, the spatial information contained in shallower features is easily corrupted and lost during the processing. As a result, the state-of-the-art general object detectors usually do not perform well in this specific application. To protect low-level special features and explore high-level semantic features at the same time, a novel framework designed for vehicle detection in aerial images is proposed in this paper. The overall architecture of the proposed framework is shown in Figure 1. Details of the proposed framework will be introduced as follows.

Section 3.1 introduces the dense waterfall residual block (DW res-block); Section 3.2 introduces the proposed multiscale receptive field attention (MRFA) module; in Section 3.3, the DW res-block is applied with MRFA module to compose an attentional dense waterfall residual block; the backbone of the proposed framework is introduced in Section 3.4.

### 3.1. Dense Waterfall Residual Block

ResNet strengthens the learning ability of the network by increasing the depth of the network. It combines earlier layers with later layers by elementwise summation, which may lead to information contamination during the process of information flow. Different from ResNet, DenseNet [44] proposed a new connection strategy. DenseNet directly connects each layer to several preceding layers by concatenation operation. According to [44], this connection strategy greatly strengthens feature reuse, improves the information flow, substantially reduces memory usage, and makes the network easier to train. Inspired by DenseNet [44], a new residual block named dense waterfall residual block (DW res-block) is proposed in this section. The proposed DW res-block keeps the setting of identity mapping in ResNet. In each block, the subsets of feature maps generated by each layer are densely concatenated with subsets of feature maps generated by preceding layers and then fed into the subsequent layers. As a result, each convolutional layer within the proposed block can obtain the subsets of all feature maps generated by the preceding layers of the same block. For example, the last layer in each block can obtain the subsets of the output of 3 preceding convolutional layers in the same block. Thus, the proposed DW res-block can preserve the shallow spatial information and extract high-level semantic information simultaneously, which is important for object detection in aerial images. Because the inner features are connected densely and the overall architecture of the proposed block looks like a waterfall, the proposed block is given the name of dense waterfall residual block (DW res-block).

**Figure 2:**
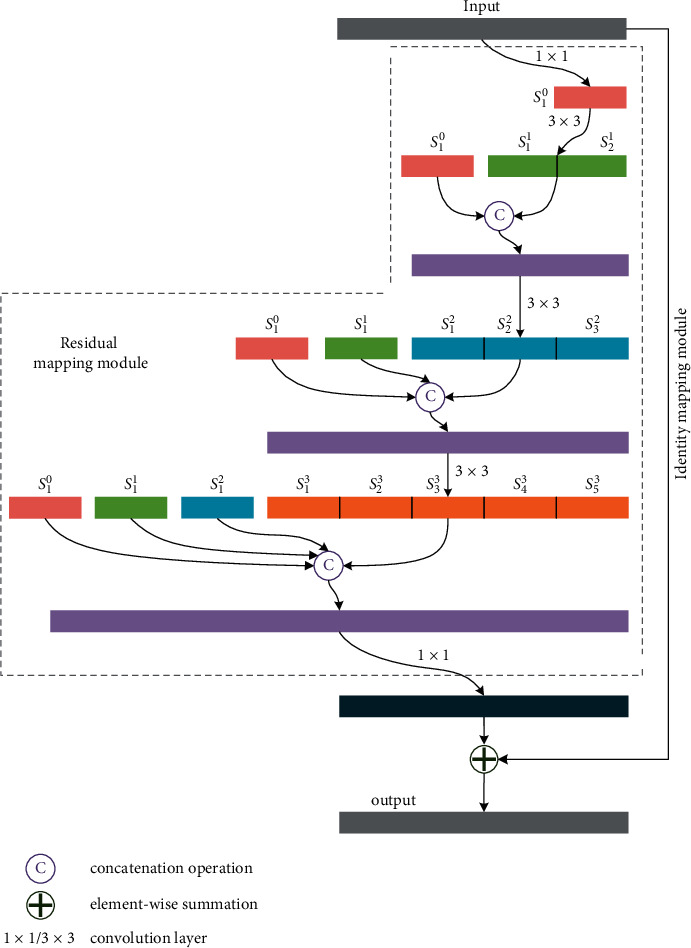
The structure of the DW res-block.

The structure of the DW res-block is shown in Figure 2. The supplementary description can be found in the box of the top-left corner. As shown in Figure 2, the rectangular boxes represent the feature maps and the width of rectangular boxes depends on the channel number of the corresponding feature map. The marks 1 × 1 and 3 × 3 nearby the connecting lines represent the convolutional layers. The width of rectangular boxes depends on the channel number of the corresponding feature map. Each convolutional layer is followed by a rectified linear unit (ReLU) function. *S*_*j*_^*i*^ denotes the subset of the corresponding feature map which is split along the channel axis. *i* denotes the number of times the subset is processed by 3 × 3 convolutional layers. *j* denotes the subset number of the corresponding feature map. The dimension of the output of the proposed block is kept the same as the input feature map.

The proposed block firstly decreases the channel dimension of the input feature map to 1/4 of origin by a 1 × 1 convolutional layer. Then, the output feature map *S*_1_^0^ which only has one subset is processed by a 3 × 3 convolutional layer, and the output feature map *S*^1^ is split into two subsets *S*_1_^1^ and *S*_2_^1^ along channel axis immediately. Following this, *S*^1^ is concatenated with *S*_1_^0^ along the channel axis, and the concatenated feature map is processed by a 3 × 3 convolutional layer to obtain the output *S*^2^. *S*^2^ is split into three subsets *S*_1_^2^, *S*_2_^2^, and *S*_3_^2^. After that *S*_1_^0^, *S*_1_^1^, and *S*^2^ are concatenated together and processed using a 3 × 3 convolution to obtain the feature map *S*^3^. Then *S*_1_^0^, *S*_1_^1^, *S*_1_^2^, and *S*^3^ are concatenated together and decreased the channel dimension to the same as the input feature map of the block by a 1 × 1 convolutional layer. Finally, the output of the 1 × 1 convolutional layer is combined with the input of the block by an elementwise summation to obtain the output of the proposed block.

(1) $$\begin{array}{c} y=f\left(x,\;\left\{W_{i}\right\}\right)+x. \end{array}$$

As shown in Figure 2, the computation process of the proposed DW res-block can be expressed as follows: where *x* and *y* denote the input and output feature maps of the proposed residual block, respectively, *W*_*i*_ denotes weights matrixes, and *f* represents the proposed residual mapping process.

### 3.2. Multiscale Receptive Filed Attention Module

**Figure 3:**
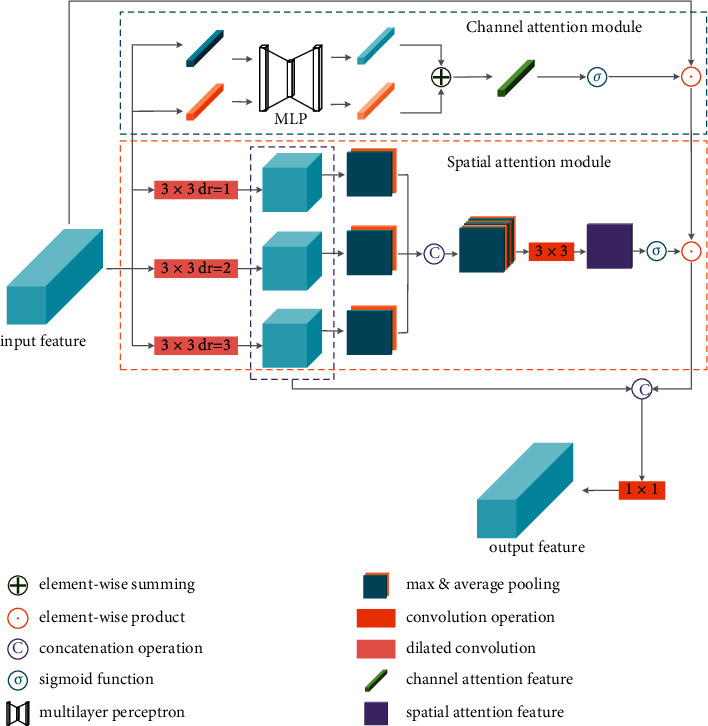
The structure of the MRFA module.

For human visual perception, the attention mechanism represents the process of human eyes concentrating on ‘what' or ‘where' in a given scene. For computer vision tasks, attention mechanism relates to the process of channel or spatial selection of a given feature map, corresponding to ‘what' and ‘where' of human visual counterpart. Due to the complex backgrounds and relatively small foreground objects, it is difficult to distinguish the foreground and background region in an aerial image. Therefore, inspired by SE-block [45], CBAM [46], and MSCA [36], to provide more information of categories and positions of foreground objects in an input aerial image, a multiscale receptive field attention (MRFA) module is proposed in this partition. Different from MSCA [36], the MRFA module generates the spatial attention map from the interspatial relationship of multiscale receptive field feature maps by using average-pooling and max-pooling operations along the channel axis. In addition, to strengthen the ability of multiscale perception, the multiscale receptive field feature maps are concatenated with the attention-weighted feature in the MRFA module. The detailed structure of MRFA is shown in Figure 3. The MRFA module generally consists of two branches: the channel attention module which is shown in the blue box and the spatial attention module which is shown in the orange box. Some supplementary description is shown in the bottom-left corner of Figure 3.

#### 3.2.1. Channel Attention Module

In the channel attention module, as shown in the blue box in Figure 3, global max-pooling (GMP) and global average-pooling (GAP) operations are both used to extract channel attention information. The GMP and GAP features are then processed using a multilayer perceptron (MLP) module. Finally, the output attention features are combined by elementwise summation. The process can be generally expressed as the following equation:

(2) $$\begin{array}{c} \omega_{c}=\;\sigma \left[MLP\left(GMP\left(X\right)\right)+MLP\left(GAP\left(X\right)\right)\right], \end{array}$$

where *X* ∈ **R**^*C*×*H*×*W*^ denotes the input feature of the attention module, and *C*,  *H*,  *W* represent the number of channels, height, and width of the feature map, respectively. *ω*_*c*_ ∈ *R*^*C*×1×1^ represents the output attention weights of the channel attention module. The *GMP* operation can be computed using the following equation:

(3) $$\begin{array}{c} GMP\left(X\right)=\underset{H,W}{\max}\left(X\right), \end{array}$$

and the GAP operation is computed as follows:

(4) $$\begin{array}{c} GAP\left(X\right)=\;\frac{1}{H\times W}\sum_{i=1}^{H}\overset{W}{\underset{j=1}{\sum }}X_{i,j}. \end{array}$$

After expanding the process of *MLP*, the whole computation process of the channel attention module can be express as

(5) $$\begin{array}{c} \omega_{c}=\;\sigma \left[W_{1}^{0}ReLU\left(W_{0}^{0}\left(CMP\left(X\right)\right)\right)+W_{1}^{1}ReLU\left(W_{0}^{1}\left(GAP\left(X\right)\right)\right)\right], \end{array}$$

where *ω*_*c*_ represents the channel attention weights, *σ* denotes the sigmoid activation function, and *W*_0_^0^ ∈ **R**^(*C*/*r*×*C*)^, *W*_1_^0^ ∈ **R**^*C*×*C*/*r*^, *W*_0_^1^ ∈ **R**^*C*×*r*/*C*^, and *W*_1_^1^ ∈ **R**^*C*×*C*/*r*^ denote the weights of *MLP*. The parameter 1/*r* denotes the reduction ratio in the bottleneck of the *MLP*. Here, *r* equates to 16. *ReLU* denotes the rectified linear unit function.

#### 3.2.2. Spatial Attention Module

Different from channel attention, the spatial attention module focuses on extracting useful position information. For the tasks of vehicle detection in aerial images, each target only occupies few pixels in the input image. Besides, the background can be more complex and confusing in aerial images. Thus, effectively applying spatial attention mechanism is difficult and important for vehicle detection in aerial images. To select the useful information from complex background areas of the input image, a multiscale receptive spatial field attention module is proposed. In the proposed module, dilated convolution is used for extracting multiscale receptive field information. Generally, the dilated convolution can effectively expand the receptive field of a network without increasing the computation cost. Besides, this strategy can effectively avoid the extraction of redundant information from the original feature map. More details will be illustrated as follows.

In the spatial attention module, multiscale receptive field information is extracted by using dilated convolution with different dilation rates. Then, the output feature maps are processed by max-pooling and average-pooling along the channel axis, respectively. Finally, the pooling feature maps are concatenated together and processed using a 3 × 3 convolution layer to generate the spatial attention weights. The calculation process can be express as

(6) $$\begin{array}{c} \omega_{s}=\sigma \left(C^{3\times 3}\left[S-pool\left(DC_{1}^{3\times 3}\left(X\right)\right),S-pool\left(DC_{2}^{3\times 3}\left(X\right)\right),S-pool\left(DC_{3}^{3\times 3}\left(X\right)\right)\right]\right), \end{array}$$

where *ω*_*s*_ ∈ **R**^1×*H*×*W*^ represents the spatial attention weights, *σ* denotes the sigmoid activation function, and *DC*_1_^3×3^ represents the dilated convolution operation (its superscript denotes the size of the convolution kernel and its subscript denotes the dilation rate). [, ] represents concatenation operation. Here, the output channel dimension of each *DC* operation will be reduced to 1/*r* of the input feature map. Here, *r* equates to 16 which keeps the same with channel attention module. *C*^3×3^ represents the convolution operation and its superscript denotes the size of the convolution kernel too. Each convolution operation and dilated convolution operation are followed by a rectified linear unit (*ReLU*) function. *S* − pool (Spatial pooling) represents max-pooling and average-pooling operations along the channel axis followed by a concatenation operation. The process of *S* − pool can be expressed as the following equations:

(7) $$\begin{array}{c} S-\text{pool}\left(X\right)=\left[Max\;{\text{Pool}}_{c}\left(X\right),\;Avg\;{\text{Pool}}_{c}\left(X\right)\right],\\ Max\;{\text{Pool}}_{c}\left(X\right)=\underset{c}{\max}X,\\ Avg\;{\text{Pool}}_{c}\left(X\right)=\frac{1}{c}\sum_{i=1}^{c}X_{i}, \end{array}$$

where *Max* *Pool*_*c*_ and *Avg* *Pool*_*c*_ represent the max-pooling and the average-pooling operations along the channel axis, and the subscript *c* denotes the channel dimension here.

#### 3.2.3. The Fusion of Channel Attention and Spatial Attention Module

In the proposed framework, to protect spatial information of shallow feature, the proposed backbone only contains 3 times downsampling operations; in other words, the total downsampling ratio of the proposed backbone is 8 (more details will be illustrated in Section 3.4). However, a small downsampling ratio leads to a small receptive field, which may be not sufficient for distinguishing vehicles from complex backgrounds in aerial images. Based on this fact, the multiscale receptive field feature maps are implemented with the attention-weighted feature map for collecting multiscale receptive field information. The process can be represented by the following equation:

(8) $$\begin{array}{c} Y=MRFA\left(X\right)=C^{1\times 1}\left[\omega_{c}⊙\omega_{s}⊙X,DC_{1}^{3\times 3}\left(X\right),DC_{2}^{3\times 3}\left(X\right),DC_{3}^{3\times 3}\left(X\right)\;\right], \end{array}$$

where *Y* ∈ **R**^*C*×*H*×*W*^ represents the output feature map, and *X* ∈ **R**^*C*×*H*×*W*^ represents the input feature map. *ω*_*c*_ ∈ **R**^*C*×1×1^ represents the channel attention weights, and *ω*_*s*_ ∈ **R**^1×*H*×*W*^ represents spatial attention weights. ⊙ denotes the elementwise product operation. *C*^1×1^ represents the 1 × 1 convolution operation for decreasing the channel number of the output feature map to the same as the channel number of the input feature map. For each input feature map, the proposed MRFA module will produce an attention-weighted feature map with the same size as the input feature map.

### 3.3. Attentional Dense Waterfall Residual Block

**Figure 4:**
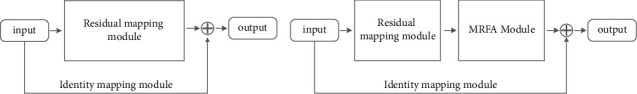
The arrangement of the MRFA module. (a) The dense waterfall residual block proposed in Section 3.2. (b) The attentional dense waterfall residual block.

The proposed MRFA module can fuse with the DW res-block introduced in Section 3.2 to compose an attentional dense waterfall residual block, which can select the informative pixels from complex background areas of the input image and strengthen the ability of multiscale perception. The general arrangement of the MRFA module and the DW res-block are shown in Figure 4. The MRFA module is placed between the residual mapping module and the identity mapping module of the proposed dense waterfall residual block. The process of attentional dense waterfall residual block can be expressed as the following equation:

(9) $$\begin{array}{c} Y=MRFA\left(f\left(X,\;\left\{W_{i}\right\}\right)\right)+X, \end{array}$$

where *MRFA* represents the process of equation (8) and *f* denotes the residual mapping introduced in equation (1).

### 3.4. The Backbone of Proposed Framework

To enlarge the receptive field, backbones such as VGG-Net, Google-Net, and ResNet involve 5 downsampling layers; as a result, the resolution of the output feature map is downsampled 32 strides relative to the resolution of the input image. This backbone design is beneficial for extracting high-level semantic information on the limited condition of memory and computation resources. However, the 32 strides' downsampling ratio will lead to the loss of spatial information, which is harmful for object localization, especially for relatively small object localization in aerial images. To solve this problem, algorithms such as YOLOv2 [17] or FPN [10] keep shallow spatial information by skip-connection or feature fusion. These methods can only alleviate the problem; it can not solve the problem. For these reasons, based on the proposed attentional dense waterfall residual block, a backbone designed for vehicle detection in aerial images is proposed. The proposed backbone preserves the spatial information from the following aspects. Firstly, inner features of the proposed residual block are connected densely which can improve the information flow between shallower and deeper layers. In addition, the identity mapping is adopted between each stage of the proposed backbone by the transition layer to further improve the information flow. The structure of the transition layer is diagrammed at the bottom of Figure 1. Secondly, the new backbone which only involves 3 times downsampling operations keeps the feature map of high resolution. Thanks to this design, the proposed backbone can extract high-level semantic information from the feature map of high resolution with alleviating loss of spatial information. Thirdly, the downprojection unit which is originally used in superresolution reconstruction [47] is applied to reduce the impact of information contamination caused by the downsampling operation. The general structure of the downprojection unit is shown in the bottom-left of Figure 1. The structure of the proposed backbone is shown in Figure 1, and details of the proposed backbone structure are listed in Table 1.

## 4. Experiments and Results

The framework proposed in this paper is evaluated on three popular public aerial datasets: VEDAI [48], UCAS-AOD [49], and DOTA [50]. In this section, these three datasets and the evaluation metrics are introduced firstly. Then, the training details of the proposed framework are illustrated. Finally, the evaluation results of the proposed framework and the efficacies of its components are analyzed and compared with other state-of-the-art detection algorithms.

### 4.1. Datasets

Deep learning based vision algorithms require large-scale labeled training data. The ground view object detection algorithms such as Faster R-CNN, YOLO, and SSD are usually trained on MS COCO [51] and PASCAL VOC [52] which contain images taken from the ground. With object detection algorithms for aerial images being widely studied, there is an increasing need for aerial image datasets. As a result, some public datasets such as NWPU VHR-10 [53], RSOD [54], VEDAI, UCAS-AOD, and DOTA are produced recently. Among these datasets, VEDIA, UCAS-AOD, and DOTA are the most commonly used datasets to evaluate vehicle detection algorithms for aerial images [55–72]. To get better comparisons with state-of-the-art algorithms, these three datasets are also used in this section to evaluate the proposed framework and its components. Some details of the three datasets used in this paper are introduced as follows.

#### 4.1.1. VEDAI Dataset

The VEDAI (Vehicle Detection in Aerial Imagery) dataset is published for the task of small vehicle detection in aerial images. Images in the VEDAI dataset are taken from the realistic and unconstrained environment. There are 4 different versions of the VEDAI dataset: LCIs (large-size color images), SCIs (small-size color images), LIIs (large-size infrared images), and SIIs (small-size infrared images). The resolutions of images in large and small versions are 1024 × 1024 and 512 × 512. The Ground Sampling Distances (GAD) of large and small versions are 12.5 cm and 25 cm, respectively. VEDAI dataset contains various backgrounds such as trees, buildings, roads, cities, and so on. The different vehicles contained in VEDAI belong to 9 categories, namely, the “plane”, “boat”, “camping car”, “car”, “pick-up”, “tractor”, “truck”, “van”, and the “other” categories. Since most of the targets in VEDAI are labeled as “small land vehicle”, i.e., “car”, “pick-up”, “tractor”, and “van”, all the targets labeled as “small land vehicles” are used to evaluate the proposed framework in this section.

#### 4.1.2. UCAS-AOD Dataset

The UCAS-AOD dataset is proposed by Patterns and Intelligent System Development Laboratory in the University of China Academy of Sciences. The dataset only contains targets from two categories: “car” and “airplane”. It contains 7482 planes in 1000 images and 7114 cars in 510 images. In this paper, targets labeled as “car” are used to evaluate the proposed framework.

#### 4.1.3. DOTA Dataset

DOTA dataset is a large-scale dataset proposed for object detection in aerial images. The resolution range of images in the DOTA dataset is from about 800 × 800 to about 4000 × 4000. The dataset contains targets from 15 categories, namely, “ship”, “plane”, “baseball diamond”, “storage tank”, “tennis court”, “swimming pool”, “ground track field”, “harbor”, “large vehicle”, “small vehicle”, “helicopter”, “roundabout”, “soccer ball field”, and “basketball court”. Because the targets from most categories in the DOTA dataset are either too large or irrelevant to the theme of this paper, only the targets labeled as “small vehicles” are employed in the evaluation of this paper.

#### 4.1.4. Image Shooting Angle and Target Scales

Because the images contained in VEDAI, UCAS-AOD, and DOTA datasets are all taken by satellites and UAVs from high altitudes, the shooting angle of these images is fixed. On the other hand, the scales of the targets used in the evaluation vary from about 30×30 pixels to 90×90 pixels. The varied scales can provide opportunities to evaluate the performance of the proposed framework on multiscale targets.

### 4.2. Evaluation Metrics

In this paper, the quantitative evaluation metrics (precision, recall, mean Average Precision (mAP), and F1-measure) are used to verify the proposed framework.

Precision is the ratio of the number of correctly detected targets to the total number of predicted examples. It is used to measure the accuracy of the proposed algorithm. It is defined as

(10) $$\begin{array}{c} \text{precision}=\frac{TP}{TP+FP}, \end{array}$$

where the (true positive) *TP* represents the number of positive examples to be correctly predicted, and (false positive) *FP* represents the number of negative examples to be predicted as a positive one.

Recall is the ratio of the number of correctly detected targets to the total number of positive examples. It is used to measure the ability to find positive examples of the proposed framework. It is defined as

(11) $$\begin{array}{c} \text{recall}=\frac{TP}{TP+FN}, \end{array}$$

where (false negative) *FN* represents the number of positive examples which are not correctly detected.

The recall and precision are generally contradictory in the same cases. Considering the negative correlation between precision and recall rate, a comprehensive evaluation metric is necessary. The F1-measure is an important metric for measuring the performance of detection algorithms, which is equally considering the recall and precision rate. The definition of the F1-measure is as follows:

(12) $$\begin{array}{c} F1-\text{measure}=\frac{2\times \text{precision}\times \text{recall}}{\text{recall}+\text{precision}}. \end{array}$$

Although F1-measure is proposed to measure the performance of object detection equally considering recall and precision, it only reflects the performance of a single point value. To solve this problem, the mAP which can reflect the global performance is proposed. It is defined as the following equations:

(13) $$\begin{array}{c} AP=\int_{0}^{1}\text{precision}\left(\text{recall}\right)d\left(\text{recall}\right),\\ mAP=\;\frac{1}{N}\sum_{i=1}^{N}AP_{i}, \end{array}$$

where *N* denotes the number of categories. *AP* measures the global performance of single category, and *mAP* measures the global performance of all categories.

### 4.3. Implementation Details

The images are processed to the resolution of 640 × 640 by sliding window cropping and padding for both training and testing stages. The experiments are performed by using an NVIDIA GeForce RTX 2080Ti GPU on TensorFlow 2.0. The weights of the proposed framework are initialized under Xavier uniform [73]. The Adam [74] optimizer and Cosine learning rate decay with an initial learning rate of 1 × 10^−4^ are used to train the proposed framework. The number of training epochs is set to 100. The learning rate decays from the beginning to the end of training with Cosine learning rate decay policy of default setting of Tensorflow 2.0.

### 4.4. Evaluation Results on VEDAI Dataset

#### 4.4.1. Comparison with Other Algorithms

**Figure 5:**
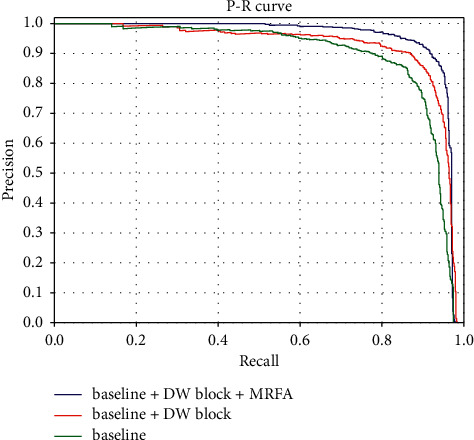
P-R curves of the comprehensive evaluation on the VEDAI dataset.

**Figure 6:**
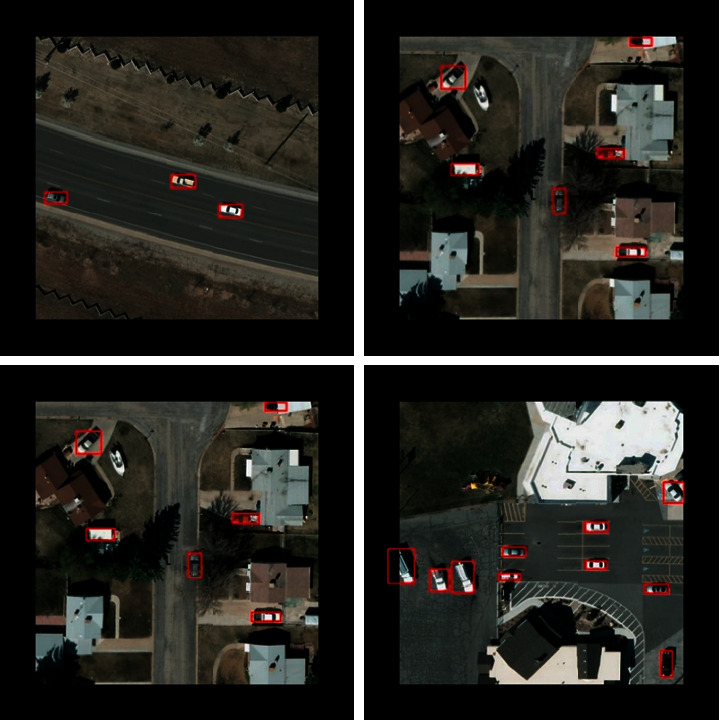
Some detection examples of the proposed framework from the VEDAI dataset.

The comparison results between the proposed framework and other state-of-the-art algorithms on the VEDAI dataset are summarized in Table 2. It can be seen that the proposed framework outperforms the existing state-of-the-art detection algorithms on the VEDAI dataset. As shown in Table 2, the proposed framework achieves an mAP of 95.13%, which is roughly 2.59% higher than L-RCNN 2020 [40] and 3.86% higher than Improved FBPN Based Detection Network [37]. The detailed results of recall, precision, F1-measure, and test time are also shown in Table 3. The P-R curves of the proposed framework are shown in Figure 5. As shown in Table 3, the proposed Dense Attentional Residual Network (Baseline + DW res-block + MRFA) achieves state-of-the-art performance on the VEDAI dataset: 89.91% for recall, 93.08% for precision, 95.13% for mAP, and 91.47% for F1-measure. To evaluate the experimental results qualitatively, some detection examples generated by the proposed framework are shown in Figure 6.

#### 4.4.2. Efficacies of the Proposed Components

To demonstrate the efficacies of the proposed DW res-block and MRFA module, in addition to the proposed DAR-Net (noted as Baseline + DW res-block + MRFA in Table 3) evaluated in the previous section, two other algorithms are evaluated using the VEDAI dataset. Firstly, a Baseline algorithm (noted as Baseline in Table 3) is implemented by keeping the backbone of DAR-Net and utilizing the regular residual block proposed in [43]. Secondly, based on the Baseline algorithm, another algorithm (noted as Baseline + DW res-block in Table 3) is implemented utilizing the proposed DW res-block instead of the regular residual block. Since the only difference between Baseline and Baseline + DW res-block is DW res-block, and the only difference between Baseline + DW res-block and Baseline + DW res-block + MRFA is MRFA, the efficacies of the proposed modules can be demonstrated by comparing the evaluation results of these three algorithms. The P-R curves of these three algorithms are shown in Figure 5. As shown in Table 3, the DW res-block contributes 2.7% improvement of mAP with only 2M increase of parameter amount, and the MRFA module has a contribution of almost 2.8% improvement of mAP with 8M increase of parameter amount. And the two proposed modules increase the processing time of 0.077s and 0.255s, respectively. The two proposed modules increase the computational complexity of the algorithm.

#### 4.4.3. Parameter Analysis

**Figure 7:**
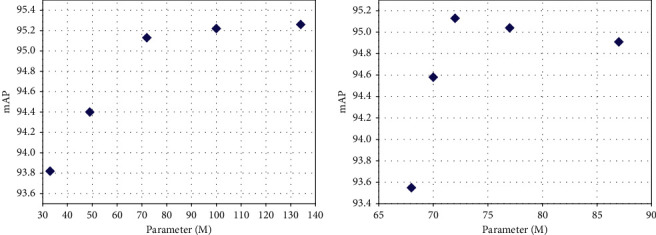
The tradeoff between model parameters amount and detection accuracy. (a) DW res-block, (b) MRFA module.

According to the experiments, the performances of the proposed DW res-block and MRFA module are both relatively sensitive to their dimension reduction ratios. Therefore, to find an effective tradeoff between model parameters amount and detection accuracy, the dimension reduction ratios of the proposed two modules are selected as hyperparameters to evaluate the parameter sensitivity of the proposed framework. The evaluation experiments are performed with the reduction ratios of DW res-block and MRFA module in the range of {1/8, 3/16, 1/4, 5/16, 3/8} and {1/64, 1/32, 1/16, 1/8, 1/4}, respectively.  Table 4 shows the evaluation results of DW res-block with different reduction ratio settings, while Table 5 demonstrates the evaluation results of MRFA module with different reduction ratio settings.  Figure 7 represents the dimension reduction ratios of the proposed two modules equal to 1/4 and 1/16 are the effective tradeoff, respectively.

### 4.5. Results on the UCAS-AOD Dataset

**Figure 8:**
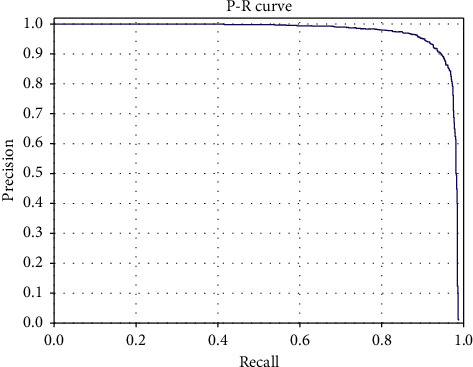
P-R curves of the proposed framework on the UCAS-AOD dataset.

**Figure 9:**
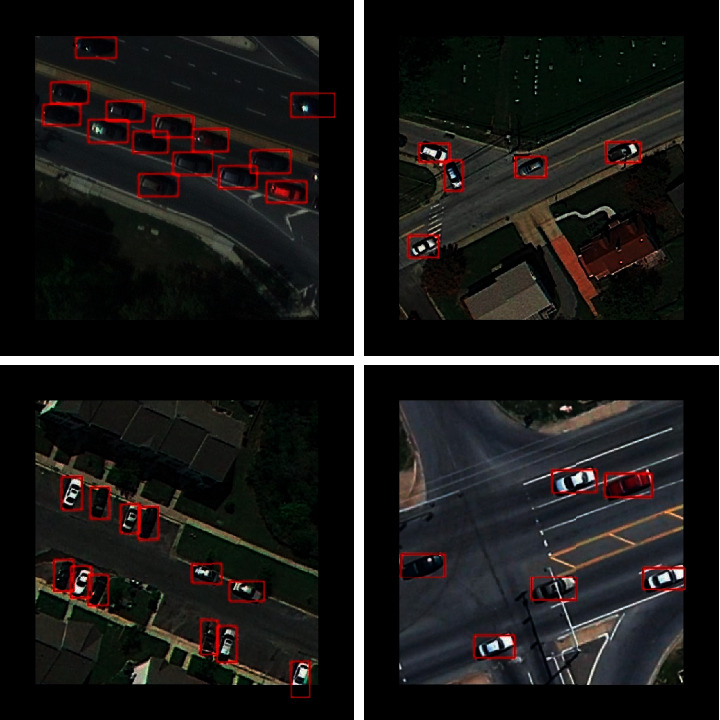
Some detection examples of the proposed framework from the UCAS-AOD dataset.

The experiment results of the proposed framework on the UCAS-AOD dataset are as follows.  Table 6 shows the comparison of performance between the proposed framework and other existing algorithms. As shown in Table 6, the proposed framework achieves an mAP of 96.78%, which outperforms the existing state-of-the-art detection algorithms on the UCAS-AOD dataset. The detailed results are shown in Table 7. It can be seen that the proposed framework achieves state-of-the-art performance on the UCAS-AOD dataset: 91.67 for recall, 94.05 for precision, 96.78 for mAP, and 92.85 for F1-measure. The P-R curve of the proposed framework on the UCAS-AOD dataset is shown in Figure 8. Some detection examples generated by the proposed framework from the UCAS-AOD dataset are shown in Figure 9.

### 4.6. Results on the DOTA Dataset

**Figure 10:**
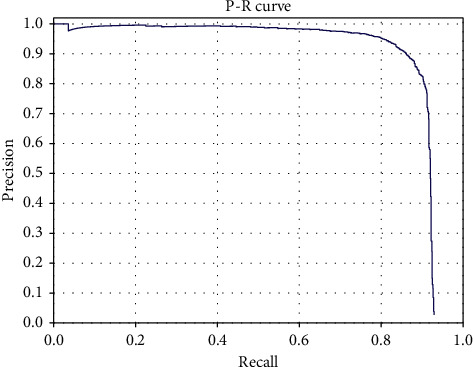
P-R curve of the proposed framework from the DOTA dataset.

**Figure 11:**
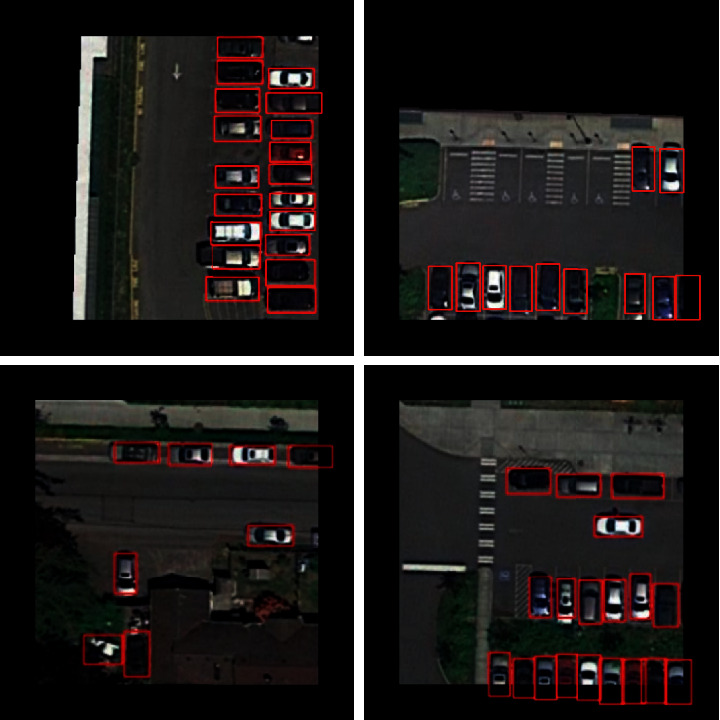
Some detection examples of the proposed framework from the DOTA dataset.

The evaluation results of the proposed framework on the DOTA dataset are as follows. The comparison of the performance of the proposed algorithm and other state-of-the-art algorithms is shown in Table 8. It can be seen that the proposed framework achieves an AP of 89.79% which is better than the performance of existing state-of-the-art detection algorithms on the DOTA dataset. The detailed evaluation results are illustrated in Table 9. As shown in Table 9, the proposed framework achieves state-of-the-art performance on the DOTA dataset: 85.97 for recall, 90.51 for precision, 89.79 for AP, and 88.18 for F1-measure. The P-R curve of the proposed framework on the DOTA dataset is shown in Figure 10. Some detection examples generated by the proposed framework from the DOTA dataset are shown in Figure 11.

## 5. Conclusions

In this paper, a novel framework named Dense Attentional Residual Network (DAR-Net) is proposed for vehicle detection in aerial images. To effectively preserve the spatial information and extract high-level semantic information at the same time, a novel residual block named dense waterfall residual block (DW res-block) is implemented in the proposed DAR-Net. To select the informative feature from the feature maps and solve the problem of the small receptive field of the proposed backbone, the multiscale receptive field attention (MRFA) module is plugged into the proposed DW res-block. Based on the DW res-block and MRFA module, a backbone designed for vehicle detection in aerial images is proposed. The proposed backbone only involves 3 times downsampling operations and extracts the semantic information from feature maps of high resolution to further preserve the spatial information. Downprojection units and transition layers are also used to reduce the impact of information loss caused by downsampling and improve the information flow, respectively. According to the experimental results, the proposed framework achieves state-of-the-art performance on VEDAI, UCAS-AOD, and DOTA datasets. The evaluation also demonstrates the efficacies of the DW res-block and the MRFA module. On the downside, object rotation still has negative effects in vehicle detection in aerial images. In the future, to improve the robustness of rotation-invariance of the proposed framework, the methods such as FourierHOG will be tried to be applied on the proposed framework, and to reduce the parameter amount without harming the performance, some solutions such as depthwise separable convolution will also be implemented in the proposed framework. Additionally, as recent researches of vehicle detection algorithms in aerial and ground view images are mutually independent, more generalized algorithms for both aerial and ground view images are worth more research for some potential applications.

## Figures and Tables

**Table 1:** The details of proposed backbone.

Layer name	Type	Output size
Convolution 1	7 × 7, stride 1	32 × 640 × 640

Downsample 1	Downprojection 1	32 × 320 × 320

Convolution 2	3 × 3, stride 1	64 × 320 × 320

Downsample 2	Downprojection 2	64 × 160 × 160

Stage 1	DW res-block × 3	64 × 160 × 160
	Transition 1	128 × 160 × 160

Downsample 3	Downprojection 3	128 × 80 × 80

Stage 2	DW res-block × 4	128 × 80 × 80
	Transition2	256 × 80 × 80

Stage 3	DW res-block × 23	256 × 80 × 80
	Transition 3	512 × 80 × 80

Stage 4	DW res-block × 3	512 × 80 × 80
	Transition 4	512 × 80 × 80

**Table 2:** Comparison with other state-of-the-art algorithms on the VEDAI dataset.

Algorithm	mAP
Faster R-CNN [55]	87.24
L-RCNN 2020 [55]	92.54
DFL 2018 [55]	90.54
FCN 2017 [56]	81.1
Faster RER-CNN 2018 [57]	82.4
Ju, et al. 2019 [58]	80.16
Improved FBPN Based Detection Network [37]	91.27
FE-YOLO 2021 [59]	89.12
DAR-Net	**95.13**

**Table 3:** The comprehensive evaluation of the proposed framework on the VEDAI dataset.

Framework	Recall	Precision	mAP	F1	Parameter (M)	Test time (s)
Baseline	85.90	85.39	89.64	85.65	62	0.043
Baseline + DW res-block	86.94	90.29	92.34	88.59	64	0.120
Baseline + DW res-block + MRFA	89.91	93.08	95.13	91.47	72	0.375

**Table 4:** The parameter analysis results of DW res-block.

Reduction ratio	Recall	Precision	mAP	F1-measure	Parameter (M)
1/8	88.57	93.87	93.82	91.15	33
3/16	90.50	93.41	94.40	91.94	49
1/4	89.91	93.08	95.13	91.47	72
5/16	91.09	94.75	95.22	92.88	100
3/8	90.06	93.38	95.26	91.69	134

**Table 5:** The parameter analysis results of MRFA module.

Reduction ratio	Recall	Precision	mAP	F1-measure	Parameter (M)
1/64	87.83	90.80	93.55	89.29	68
1/32	88.43	91.83	94.58	90.10	70
1/16	89.91	93.08	95.13	91.47	72
1/8	90.21	90.48	95.04	90.34	77
1/4	91.84	90.76	94.91	91.30	87

**Table 6:** Comparison with other state-of-the-art algorithms on the UCAS-AOD dataset.

Algorithm	AP
R-DFPN 2018 [28]	82.5
Improved Faster R-CNN [60]	83.0
DRBox 2017 [61]	85.0
*O* ^2^-DNet 2016 [62]	86.72
P-RSDet 2020 [35]	87.36
R–FCN 2016 [35]	89.3
Deformable R–FCN [35]	91.7
S2ARN 2019 [61]	92.2
FADet 2019 [63]	92.72
RetinaNet-H 2019 [64]	93.6
R3Det 2019 [64]	94.14
A2RMNet [60]	94.65
SCRDet++ 2020 [65]	94.97
PolarDet 2020 [66]	94.96
ICN 2018 [29]	95.67
UCAS + NWPU + VS-GANs 2019 [67]	96.12
Improved FBPN Based Detection Network [37]	96.18
FE-YOLO 2021 [59]	90.85
DAR-Net	**96.78**

**Table 7:** Detailed results of proposed framework on the UCAS-AOD dataset.

Algorithm	Recall	Precision	AP	F1-measure
DAR-Net	91.67	94.05	96.78	92.85

**Table 8:** Comparison with other state-of-the-art algorithms on the DOTA dataset.

Algorithm	AP
LR-CNN 2020 [68]	56.09
Yang et al. 2018 [37]	61.16
RoI Transformer [69]	68.81
The Light-Head R-CNN OBB + *W*/FPN [69]	70.15
Faster R-CNN Adapted 2018 [70]	74.9
DYOLO Module B 2018 [70]	76.0
SSD Adapted 2018 [70]	76.3
DFRCNN 2018 [71]	76.5
PolarDet [66]	78.53
DSSD 2017 [21]	79.0
DYOLO Module A 2018 [70]	79.2
RefineDet 2018 [70]	80.0
Ju, et al. 2019 [58]	88.63
Faster R-CNN with MSCA [36]	26.85
SSD with MSCA [36]	23.99
HyNet (Hy-64-ResNet-50) [72]	64.58
Improved FBPN Based Detection Network [37]	88.76
DRA-net	**89.79**

**Table 9:** Detailed results of proposed framework on the DOTA dataset.

Algorithm	Recall	Precision	AP	F1-measure
DAR-Net	85.97	90.51	89.79	88.18

## Data Availability

The data supporting this study were taken from previously reported studies and datasets, which have been cited. The processed data are available from the corresponding author upon request.
